# Socio-demographic predictors of insecticide-treated bed net ownership and utilization for protection against malaria by rural community members across five regions of Mainland Tanzania

**DOI:** 10.1186/s12936-026-05926-9

**Published:** 2026-05-07

**Authors:** Gervas A. Chacha, Misago D. Seth, Salehe S. Mandai, Daniel A. Petro, Daniel P. Challe, Angelina J. Kisambale, Rule Budodo, Rashid A. Madebe, Ruth B. Mbwambo, Catherine Bakari, Dativa Pereus, Sijenunu Aaron, Daniel Mbwambo, Abdallah Lusasi, Samuel Lazaro, Celine I. Mandara, Deus S. Ishengoma

**Affiliations:** 1 National Institute for Medical Research, Dar es Salaam, Tanzania; 2 Ifakara Health Institute, Dar es Salaam, Tanzania; 3 University of Dar es Salaam, Dar es Salaam, Tanzania; 4 National Institute for Medical Research, Tanga Research Centre, Tanga, Tanzania; 5 Muhimbili University of Health and Allied Sciences, Dar es Salaam, Tanzania; 6 National Malaria Control Programme, Dodoma, Tanzania

**Keywords:** Malaria, *Plasmodium falciparum*, Insecticide-treated bed nets, Tanzania

## Abstract

**Background:**

Despite decades of control efforts. malaria burden in Tanzania remains high, with marked heterogeneity in transmission intensity across regions. Insecticide-treated bed nets (ITNs) are a core malaria intervention and are distributed through multiple channels in Tanzania to promote equitable access and use, yet disparities in ITNs ownership and use persist. This study evaluated socio-demographic predictors of ITNs ownership and use among rural communities from five regions with varying malaria endemicity.

**Methods:**

A community-based cross-sectional survey covering individuals aged ≥ 6 months was conducted from July to August 2023 in 15 villages across five districts from five regions of Mainland Tanzania (Kagera, Kigoma, Njombe, Ruvuma, and Tanga). Data on demographics, malaria prevention practices, anthropometrics and socio-economic status (SES) were collected using structured questionnaires installed in tablets, run with Open Data Kit (ODK) software. Socio-demographic predictors of ITNs ownership and use were assessed using logistic regression analysis. The results were reported as crude (cOR) and adjusted odds ratios (aOR) with 95% confidence intervals (CI) and a p-value < 0.05 was considered statistically significant.

**Results:**

Among the 10,228 enrolled participants, 7939 (77.6%) and 7899 (77.2%) reported owning and using ITNs, respectively. ITNs ownership and use varied significantly across districts (p < 0.001), with the highest rates observed in Nyasa (Ruvuma) and the lowest in Kyerwa (Kagera). Females had higher odds of both ITNs ownership and use than males (aOR = 1.27, 95% CI 1.12–1.45, p < 0.001 for both outcomes). Under-fives were more likely to own (aOR = 1.83, 95%CI 1.56–2.15, p < 0.001) and use ITNs (aOR = 2.26, 95%CI 1.62–3.15, p < 0.001) than adults. Participants from Nyasa (Ruvuma), Ludewa (Njombe), Muheza (Tanga) and Buhigwe (Kigoma) districts exhibited higher odds of ITNs ownership and use compared to those from Kyerwa (Kagera) (p < 0.001). Higher education attainment and household SES were independently associated with increased ITNs ownership and use (p < 0.001).

**Conclusion:**

Although ITNs ownership and use were relatively higher across the surveyed communities, coverage remained below the national target of 80% (projected for 2023). Higher ITNs ownership and use were reported among females, under-fives, participants with higher education and those from households with high SES. Disparities by sex, age groups, household SES and education status persist and should be explicitly addressed through ITNs distribution strategies to enable equitable access and use of ITNs across all population groups to expedite progress toward malaria elimination in Tanzania.

**Supplementary Information:**

The online version contains supplementary material available at 10.1186/s12936-026-05926-9.

## Background

Despite sustained malaria control efforts led by the National Malaria Control Programme (NMCP), malaria remains a major health challenge in Tanzania [1]. Insecticide-treated bed nets (ITNs) are the cornerstone of malaria control and together with other interventions have contributed to a marked decline of malaria burden in Mainland Tanzania, with parasite prevalence among under-fives dropping from 18.1% in 2008 to 8.1% in 2022 [2, 3]. Since 2004, Tanzania has implemented multiple ITNs distribution strategies including the Tanzania National Voucher Scheme (TVNS) that provided ITNs to pregnant women and infants [4, 5], mass distribution campaigns targeting under-fives and other high-risk groups [6, 7], and school net programmes (SNPs) operating through primary schools [6, 8, 9] which initially covered three perennial endemic regions in three consecutive years from 2013 to 2015 [10] and was later in 2017 expanded geographically to cover over half of Tanzania Mainland [8, 11]. These initiatives aimed to achieve and sustain universal ITNs coverage to the rate of at least one ITN for every two people and reaching a coverage of 80% by 2023 and 85% by 2025 [12, 13].

Besides ITNs distribution programs, behaviour change communication (BCC) campaigns have been introduced to promote ITNs ownership and consistent use [14–17]. They disseminate key messages featured with widely recognized slogans such as “Malaria haikubaliki” (Malaria is not acceptable) and “Ziro malaria inaanza na mimi” (Zero malaria starts with me) which advocate malaria risk awareness and fostering acquisition and proper use of ITNs to reduce malaria transmission [16, 18]. Despite the extensive efforts, operational constrains, behavioural barriers and emerging challenges such as insecticide resistance, reduced net durability and misuse continue to limit the effectiveness of ITNs [13, 19–23].

Due to substantial spatial heterogeneity in malaria transmission, the Tanzania NMCP has transitioned from uniform “one size fits all” control strategy to a stratified, burden-based approach that tailors malaria interventions to local epidemiological context [12, 24–26], and the estimation of ITNs access and quantification for programmatic action are currently conducted at council level [1, 27, 28]. However, these efforts largely rely on infrequent nation surveys such as malaria indicator survey or demographic and health survey, which limit the ability to monitor socio-demographic disparities and to timely respond to emerging vulnerability. This study assesses the socio-demographics determinants of ITN ownership and use across rural communities in five regions of Mainland Tanzania spanning multiple transmission strata, providing the novel insights on population-specific patterns of ITNs access and utilization. These findings are critical for refining targeted ITNs distributions strategies, improving equity in ITNs access as well as strengthening stratified malaria control efforts as Tanzania advances towards malaria elimination.

## Methods

### Study design and sites

**Fig. 1:**
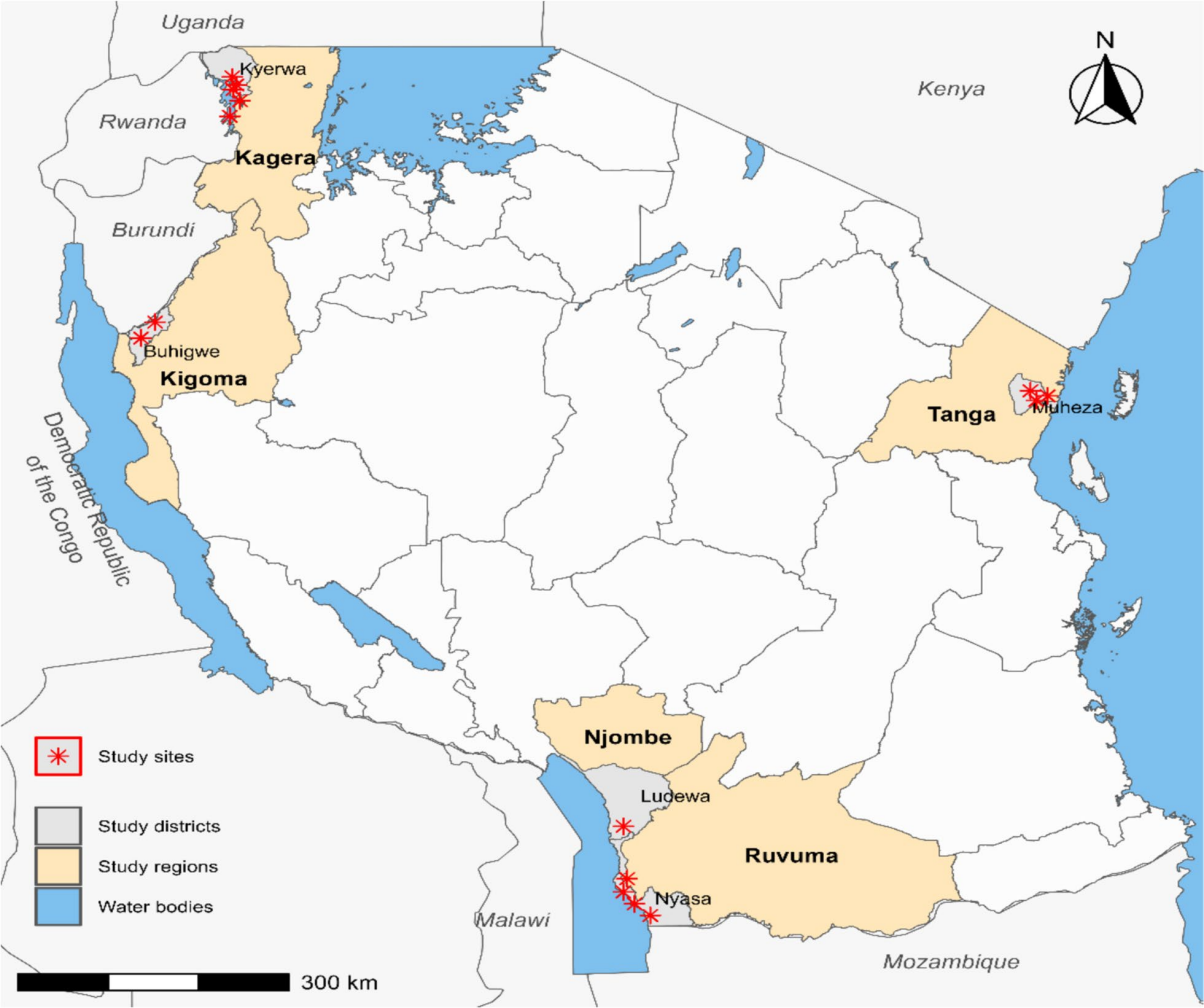
A map of Tanzania showing the regions, districts and study sites/villages that were covered in the community-based cross-sectional surveys in 2023

This study utilized data collected from the community-based cross-sectional surveys (CSS) conducted in five regions of Tanzania Mainland with varying malaria transmission intensities as previously reported [29, 30]. The data were collected by the project on Molecular Surveillance of Malaria in Mainland Tanzania (MSMT) which involved both health facilities and community surveys. The regions involved in CSS include two each from high (Tanga and Ruvuma) and moderate (Kagera and Kigoma), and one region (Njombe) from low transmission strata. In each region, one district and varying numbers of villages were selected. In Tanga region, the survey covered three villages (Magoda, Mamboleo and Mpapayu) from Muheza district [31]. In Kagera region, five villages were covered (Kitwechenkura, Nyakabwera, Rubuga, Kitoma and Ruko) and all were from Kyerwa district as previously described [30], while in Kigoma, the surveys were done in two villages (Nyankoronko and Kigege) both from Buhigwe district. In Ruvuma, the study was conducted in four villages (Lundo, Lipingo, Ngindo and Chiulu) from Nyasa district and in Njombe region, one village (Kipangala) from Ludewa district was covered (Fig. 1). The majority of individuals from these villages are served by dispensaries that are involved in the longitudinal component of the MSMT project since 2022, aiming to monitor parasite populations and malaria patterns in these regions, as described elsewhere [29, 32, 33].

### Study population and recruitment process

Data used in this study were collected in the community surveys which aimed to recruit at least 30% all individuals aged ≥ 6 months residing households located within study villages which were registered during the census surveys conducted before the CSS. In the targeted communities, household registration was done earlier through the census surveys conducted by the MSMT project team before the CSS, as described earlier [29, 30]. The decision to target 30% of the community members was informed by prior studies which showed that such sample size provided a better representation of the communities and generated findings comparable to community surveys [29, 30]. For the CSS, the inclusion criteria included individuals aged six months and above, residing in the registered households within the study villages and providing an informed consent. Individuals from villages whose households were not registered and those who declined to provide an informed consent were not recruited in this study. All individuals from registered households within the study villages were informed about CSS and invited to participate willingly in the surveys as described elsewhere [29–31]. Repeated visits were done where feasible to minimize non-response rates, and absent individuals were allowed to visit the survey team in nearby villages within districts where CSS spanned multiple villages.

### Data collection procedures

This study utilized data collected in both census and CSS as previously described [38, 39]. In summary, prior to the CSS, census surveys were conducted to collect demographic data, register households, obtain household socio-economic status (SES) as well as environmental, together with land use and geographic information system data. The census also involved enumeration of the community members and their households and providing them with unique identification numbers (IDs). During CSS, all participants were identified based on the IDs provided, and data collection proceeded as described earlier [29, 30]. In brief, the data collection involved verifying participants’ identity, assigning study-specific IDs for the CSS and issuing the CSS-specific registration cards. Then, all verified and registered participants were invited to provide consent or assent (for participants aged 7–17 years), followed by interviews on socio-demographics and malaria prevention practices, including ITN ownership and use. ITNs ownership was operationally defined as participant reported household possession of at least one ITN at the time of the survey. ITNs use was defined as self-reported sleeping under an ITN in the previous night before the survey. Thereafter, participants were directed to the next sections for anthropometric measurements, followed by the laboratory section for screening for malaria parasites using rapid diagnostic tests (RDTs) and collection of blood samples as blood films (thin and thick smears for malaria diagnosis by microscopy) and dried blood spots on Whatman filter paper for further laboratory analysis. Lastly, participants visited the clinical section where they were assessed by the study clinicians to collect data on history of illness and any treatment received within two weeks before surveys, followed by physical and clinical examination, as well as treatment in case the RDT was positive for malaria or for any other illnesses, which were managed accordingly [29].

### Data management and analysis

The data were collected using questionnaires prepared and configured using Open Data Kit (ODK) software installed in tablets. Collected data were instantly transferred to the central server at the National Institute for Medical Research (NIMR), in Dar es salaam for integration and quality checks. Data discrepancy and unexpected values were highlighted for rejection or prompted correction from the field team. Additional data cleaning was done in Excel and later transferred to STATA version 13 (STATA Corp Inc., TX, USA) for final cleaning and analysis. Descriptive analysis was performed to provide baseline information and demographic features of the study populations. The relationships between categorical variables were assessed using chi-square test, and the results were presented in texts, tables and figures. Multilevel logistic regression was used to assess the association of ITNs ownership and use and other covariates such as age groups, sex, education levels, occupation, family size and household SES. Variables with p < 0.25 in the univariate analysis were fitted into multivariate models. In the hierarchical model-building strategy. Multilevel logistic regression was used to assess clustering of ITNs ownership and use. The null model assessed clustering across nesting levels (households, villages and districts). Random intercepts were retained only at the household level (intracluster-correlation coefficient (ICC) > 0.70 for both outcomes [34–36]. Model I adjusted for individual-level covariates (sex, age group, education level and occupation), while the final model (Model II) incorporated both individual and household factors, including family size and household SES, as described previously [29]. Selection of covariates was informed by the biological plausibility and existing literature, and their collinearity was assessed using the variance inflation factor (VIF). Only variables with VIF values below accepted threshold (< 5) were retained in the models to avoid multicollinearity. Model fitness was evaluated using the Akaike Information Criterion (AIC) and Likelihood ratio tests [36–39]. Principal component analysis (PCA) was used to determine the SES of the households as previously described [40]. Some variables which were considered in the PCA were: source of power for light, ownership of radio, motorcycle, mobile phone, bicycle and livestock, number of sleeping rooms per house, and the number of acres of land cultivated by a family (Supplementary Table 1). All variables were coded as binary or categorical indicators and standardized prior to analysis. The first component with eigenvalues > 1 in household socio-economic characteristics, was retained as SES index, which were then ranked to create the household wealth index categorized into three SES groups; low, moderate and high using cut-off points corresponding to 40:40:20 distribution [40, 41]. The association between variables was reported as crude (cOR) or adjusted odds ratios (aOR) with 95% confidence intervals (CIs), and a p-value ≤ 0.05 was considered statistically significant.

## Results

### Baseline characteristics of the study participants

This CSS was conducted from July to August 2023 and enrolled 10,228 individuals from rural communities (with or without symptoms of malaria) across five districts in five regions of Mainland Tanzania. Females constituted 60.3% (n = 6163/10228) of the participants, while males accounted for 39.7% (n = 4065/10228), and their overall median age was 14 years (Interquartile range (IQR) 7–38). Adults aged ≥ 15 years represented 47.9% (n = 4899/10228), followed by school-children (5–< 15 years) who accounted for 35.6% (n = 3640/10228), and under-fives constituted 16.5% of the participants (n = 1689/10228). Among the five districts, the largest proportion of the participants were from Kyerwa (43.6%, n = 4454/10228), followed by Nyasa (24.0%, n = 2455/10228), Buhigwe (14.2%, n = 1453/10228), Muheza (12.3%, n = 1255/10228) and Ludewa (6.0%, n = 611/10228). A history of fever in the past 48 h prior to the survey was reported by 20.6% of the participants (n = 2102/10228), while 2.3% (n = 238/10228) had fever at presentation. Most of the participants were involved in farming (39.8%, n = 2565/6446) and 37.0% (n = 2411/6524) had completed primary education. With respect to SES, 40.9% (n = 3953/9664) of the participants were from households with high SES, 34.2% (n = 3302/9664) from those with moderate, and 24.9% (n = 2409/9664) from households with low SES (Table 1)**.**

**Table 1:** Baseline characteristics of study participants

Characteristics	Ludewa	Buhigwe	Muheza	Kyerwa	Nyasa	Total
Enrolled, N (%)	611 (6.0)	1453 (14.2)	1255 (12.3)	4454 (43.6)	2455 (24.0)	10,228
Sex, n (%)						
Female	362 (59.3)	914 (62.9)	720 (57.4)	2641 (59.3)	1526 (62.2)	6163 (60.3)
Male	249 (40.8)	539 (37.1)	535 (42.6)	1813 (40.7)	929 (37.8)	4065 (39.7)
Age in years, Median (IQR)	14 (7–43)	12 (7–26)	14 (8–39)	14 (7–36)	18 (9–45)	14 (7–38)
Age groups, n (%)						
< 5 years	96 (15.7)	260 (17.9)	160 (12.7)	835 (18.8)	338 (13.8)	1689 (16.5)
5–< 15 years	219 (35.8)	672 (46.2)	493 (39.3)	1471 (33.0)	785 (32.0)	3640 (35.6)
15 + years	296 (48.5)	521 (35.9)	602 (48.0)	2148 (48.2)	1302 (54.3)	4899 (47.9)
History of fever past 48 h, n (%)						
Yes	72 (11.8)	338 (23.3)	246 (19.6)	1342 (30.1)	104 (4.2)	2102 (20.6)
No	539 (88.2)	1115 (76.7)	1009 (80.4)	3112 (69.9)	2351 (95.8)	8126 (79.4)
Fever at presentation (≥37.5 °C), n (%)						
Yes	5 (0.8)	25 (1.7)	21 (1.7)	136 (3.1)	51 (2.1)	238 (2.3)
No	606 (99.2)	1428 (98.3)	1234 (98.3)	4318 (96.9)	2404 (97.9)	9990 (97.7)
Education level, n (%)						
None	12 (3.0)	92 (9.3)	40 (5.9)	566 (20.5)	75 (4.5)	785 (12.0)
Incomplete primary	107 (26.3)	362 (36.4)	225 (32.9)	827 (30.0)	507 (30.2)	2028 (31.1)
Completed primary	199 (48.9)	266 (26.7)	263 (38.5)	905 (32.8)	778 (46.3)	2411 (37.0)
Secondary or above	21 (5.2)	10 (1.0)	28 (4.1)	75 (2.7)	87 (5.2)	221 (3.4)
Studying	68 (16.7)	265 (26.6)	128 (18.7)	386 (14.0)	232 (13.8)	1079 (16.5)
Occupation, n (%)						
Farmer	232 (41.9)	60 (16.0)	6 (6.1)	1634 (41.5)	633 (42.7)	2565 (39.8)
Students	155(28.0)	137 (36.6)	32 (32.7)	1061 (26.9)	469 (31.7)	1854 (28.8)
Children	147 (26.5)	160 (42.8)	52 (53.1)	1180 (30.0)	305 (20.6)	1844 (28.6)
Others	20 (3.6)	17 (4.6)	8 (8.2)	64 (1.6)	74 (5.0)	183 (2.8)
Socio-economic status, n (%)						
High	253 (42.6)	598 (45.2)	833 (67.7)	1102 (26.7)	1167 (48.9)	3953 (40.9)
Moderate	287 (48.3)	439 (33.2)	274 (22.2)	1516 (36.7)	786 (32.9)	3302 (34.2)
Low	54 (9.1)	286 (21.6)	124 (10.1)	1509 (36.6)	436 (18.2)	2409 (24.9)

*N* Total participants, *n* Number of observed participants, *IQR* Inter-quantile range, *°C* degree Celsius

### Insecticide treated bed net ownership among study participants

**Fig. 2:**
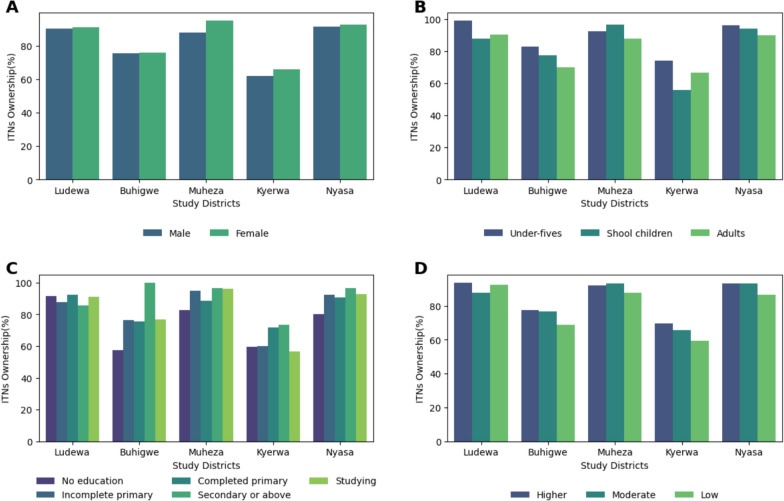
Distribution of ITNs ownership across participant by sex (upper left), age groups (upper right), education level (lower left), and socio-economic status (lower right), in the five study districts

Overall, 77.6% (n = 7939/10228) of the participants reported owning an ITNs (Table 2). Ownership rates varied significantly across districts (p < 0.001), ranging from 92.1% (n = 2262/2455) in Nyasa and to 64.4% (n = 2867/4454) in Kyerwa. Females consistently reported higher ITNs ownership than males, with statistically significant differences observed in Muheza (p < 0.001) and Kyerwa district (p = 0.006) (Fig. 2A). ITNs ownership also varied by age groups (overall p ≤ 0.006), under-fives demonstrated higher ownership across districts, except in Muheza where school-children had the highest ownership. Adults had comparatively lower ITNs ownership in Buhigwe, Muheza, and Nyasa districts, while school children had the lowest ownership of INTs in Ludewa and Kyerwa (Fig. 2B). Participants reporting fever in the past two days had lower ITNs ownership than those without recent fever, except in Nyasa, although this difference was statistically significant only in Muheza (p = 0.004). However, ITNs ownership was statistically similar among participants with or without fever at presentation (Supplementary Table 2 A). Participants with higher education levels were generally more likely to own ITNs across all districts. However, in Ludewa higher levels were observed among subgroups with no education, complete primary education or studying (Fig. 2C). Across all districts, ITNs ownership increased with household SES, with the lowest ownership was observed among individuals from households with low SES, except in Ludewa, where those from households with moderate SES had the lowest ownership (Fig. 2D).

**Table 2:** Insecticide-treated nets ownership and use in the five surveyed districts

	Insecticide-treated nets ownership	Insecticide-treated nets use
Variable		
Overall, n (%)	7939 (77.6)	7899 (77.2)
Sex, n (%)		
Female	4864 (78.9)	4842 (78.6)
Males	3075 (75.7)	3057 (75.2)
p-value	< 0.001	< 0.001
Age groups, n (%)		
< 5 years	1401 (83.0)	1398 (82.7)
5–< 10 years	2749 (75.5)	2736 (75.2)
15 + years	3789 (77.3)	3765 (76.9)
p-value	< 0.001	< 0.001
Study district, n (%)		
Ludewa	555 (90.8)	555 (90.8)
Buhigwe	1101 (75.8)	1094 (75.3)
Muheza	1154 (92.0)	1145 (91.2)
Nyasa	2262 (92.1)	2256 (91.9)
Kyerwa	2867 (64.4)	2849 (64.0)
p-value	< 0.001	< 0.001
Education level, n (%)		
None	494 (62.9)	492 (62.7)
Incomplete primary	1551 (76.5)	1536 (75.7)
Completed primary	1975 (81.9)	1960 (81.3)
Secondary or above	194 (87.8)	194 (87.8)
Studying	822 (76.2)	821 (76.1)
p-value	< 0.001	< 0.001
Socio-economic status, n (%)		
High	3327 (77.9)	3312 (83.8)
Moderate	2569 (77.8)	2555 (77.4)
Low	1628 (67.6)	1618 (67.2)
p-value	< 0.001	< 0.001

### Insecticide treated bed net use among study participants

**Fig. 3:**
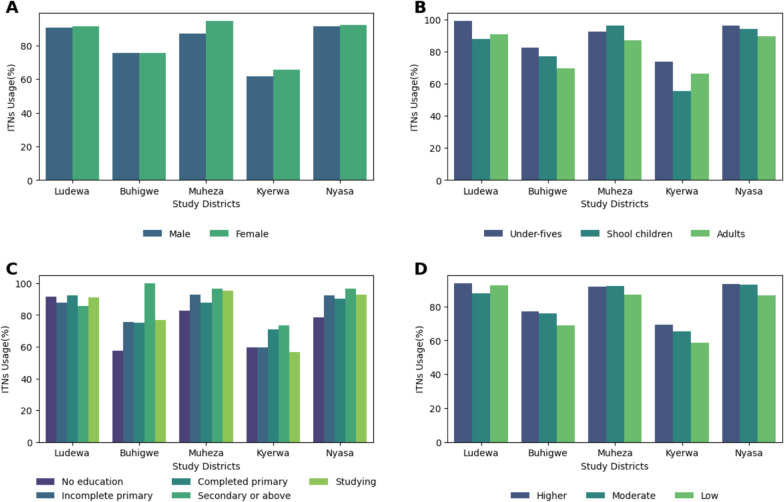
Distribution of ITNs use across participant characteristics, including sex (upper left), age groups (upper right), education level (lower left), and socio-economic status (lower right), in the five study districts

Of the study participants, 77.2% (n = 7899/10288) reported using ITNs (Table 2) and the highest use of nets was reported in Nyasa (91.9%, n = 2256/2455) while the lowest was in Kyerwa (64.0%, n = 2899/4454), with significant variation across districts (p < 0.001). Females reported higher ITNs use in all districts except Buhigwe, where use was similar among male and female participants (p = 0.917) (Fig. 3A). ITN use varied significantly by age groups across districts (p ≤ 0.006), with higher use among under-fives in most of the districts, except in Muheza where school-children reported higher use. Lower use of ITNs was observed among schoolchildren in Kyerwa and Ludewa, and among adults in Buhigwe, Muheza and Nyasa districts (Fig. 3B). Participants with recent fever (past 48 h) reported lower ITNs use, but the differences were statistically significant in Kyerwa district only (p = 0.004). In all districts (combined), ITNs use did not differ among participants with or without fever at presentation, but higher use was reported among febrile participants in Ludewa and Buhigwe districts (Supplementary Table 2B). ITNs use was higher among participants with higher education level in most of the districts (p < 0.001). However, in Ludewa, peak ITNs use was observed among participants with no education status, completed primary or studying (Fig. 3C). ITNs use was higher among study participants living in household with higher SES in most of the districts and use of ITNs was significantly lower among participants from households with low SES (Fig. 3D).

### Factors influencing ITNs ownership and use among study participants

In multivariate logistic regression analyses, females had higher odds of both ITNs ownership and use compared with males (aOR = 1.27, 95% CI 1.12–1.45, p < 0.001 for both outcomes). Under-fives were more likely to own (aOR = 1.83, 95%CI 1.56–2.15, p < 0.001) and use ITN (aOR = 2.26, 95%CI 1.62–3.15, p < 0.001) than adults, while no significant differences in ownership and use were observed among school-children and adults. Compared to Kyerwa, participants from Ludewa, Muheza, Nyasa and Buhigwe had significantly higher odds (aOR ≥ 1.55 with varying 95% CI, p < 0.001) of ITNs ownership and use. Higher education attainment was strongly associated with higher odds of ITNs ownership and use. Using participants with no formal education as the reference group, higher odds of ITNs ownership and use were observed among students (ownership: aOR = 1.21, 95%CI 0.90–1.62, p = 0.200; use: aOR = 1.27, 95%CI 1.02–1.58, p = 0.031), those with incomplete primary education (ownership: aOR = 1.27, 95%CI 1.02–1.59, p = 0.036; use: aOR = 1.26, 95%CI 1.05–1.53, p = 0.016), participated who completed primary education (ownership: aOR = 1.60, 95%CI 1.33–1.94, p < 0.001; use: aOR = 1.55, 95%CI 1.29–1.88, p < 0.001), and those with secondary education or higher levels (ownership: aOR = 2.17, 95%CI 1.39–3.41, p = 0.001; use: aOR = 2.21,95%CI 1.41–3.46, p = 0.001). Similarly, participants from households with moderate and high SES had higher odds of owning [aOR = 1.31, 95%CI 1.13–1.53, p = 0.001(for moderate SES) and aOR = 1.61, 95%CI 1.36–1.90, p < 0.001 (for high SES)] and use of ITNs [aOR = 1.31, 95%CI 1.12–1.52, p = 0.001(for moderate SES) and aOR = 1.60, 95%CI 1.36–1.89, p < 0.001(for high SES)] compared to those from households with low SES (Table 3).

**Table 3:** Factors associated with insecticide-treated bed nets ownership and use

	Insecticide-treated bed net ownership				Insecticide-treated bed net use			
Variable	cOR, 95% CI	p-value	aOR, 95% CI	p-value	cOR, 95% CI	p-value	aOR, 95% CI	p-value
Sex								
Female	1.21 (1.10—1.32)	< 0.001	1.27 (1.12—1.45)	< 0.001	1.21 (1.10—1.33)	< 0.001	1.27 (1.12—1.45)	< 0.001
Males (reference)	1		1		1		1	
Age groups								
< 5 years	1.43 (1.23—1.64)	< 0.001	1.83 (1.56—2.15)	< 0.001	1.45 (1.25—1.67)	< 0.001	2.26 (1.62—3.15)	< 0.001
5–< 10 years	0.90 (0.82—1.01)	0.050	1.04 (0.84—1.28)	0.708	0.91 (0.82—1.01)	0.007	1.02 (0.78—1.34)	0.864
15 + years (reference)	1		1		1		1	
Study district								
Ludewa	5.49 (4.14—7.27)	< 0.001	4.52 (3.18—6.41)	< 0.001	5.58 (4.21—7.40)	< 0.001	4.64 (3.27—6.58)	< 0.001
Buhigwe	1.73 (1.51—1.98)	< 0.001	1.55 (1.31—1.85)	< 0.001	1.72 (1.50—1.96)	< 0.001	1.57 (1.32—1.86)	< 0.001
Muheza	6.32 (5.11—7.82)	< 0.001	5.50 (4.09–7.39)	< 0.001	5.86 (4.78—7.20)	< 0.001	4.96 (3.74—6.57)	< 0.001
Nyasa	6.49 (5.53—7.61)	< 0.001	5.02 (4.09—6.15)	< 0.001	6.39 (5.46—7.47)	< 0.001	4.92 (4.03—6.01)	< 0.001
Kyerwa (reference)	1		1		1		1	
Education level								
Secondary or above	4.23 (2.76—6.49)	< 0.001	2.17 (1.39—3.41)	0.001	4.28 (2.79—6.56)	< 0.001	2.21 (1.41—3.46)	0.001
Completed primary	2.67 (2.23—3.19)	< 0.001	1.60 (1.33—1.94)	< 0.001	2.59 (2.17—3.09)	< 0.001	1.55 (1.29—1.88)	< 0.001
Incomplete primary	1.92 (1.60—2.29)	< 0.001	1.27 (1.02—1.59)	0.036	1.86 (1.56—2.22)	< 0.001	1.26 (1.05—1.53)	0.016
Studying	1.88 (1.54—2.30)	< 0.001	1.21 (0.90—1.62)	0.200	1.89 (1.55—2.32)	< 0.001	1.27 (1.02—1.58)	0.031
None (reference)	1		1		1		1	
Socio-economic status								
High	2.55 (2.26—2.88)	< 0.001	1.61 (1.36—1.90)	< 0.001	2.53 (2.24—2.85)	< 0.001	1.60 (1.36—1.89)	< 0.001
Moderate	1.68 (1.49—1.89)	< 0.001	1.31 (1.13—1.53)	0.001	1.67 (1.49—1.88)	< 0.001	1.31 (1.12—1.52)	0.001
Low (reference)	1		1		1		1	

*cOR* Crude odds ratio, *aOR* Adjusted odds ratio

The analysis of random effects analysis revealed strong household-level clustering, with over 70% of total variance in ITNs ownership and use attributable to household-level factors (Null Model). The last model (Model II in both ITNs ownership and use) with lowest AIC and likelihood ratio was considered the best fit for predicting the association between independent variables and ITNs ownership and use (Table 4).

**Table 4:** Random effect model and comparison of best fit for predictors associated with ITNs ownership and use for malaria protection

Parameters	Insecticide-treated net ownership			Insecticide-treated net use		
	Null model	Model I	Model II	Null model	Model I	Model II
Cluster-level variance (SE)	8.44(0.775)	5.30(0.778)	5.13(0.746)	8.11(0.733)	5.55(0.813)	5.36(0.777)
ICC	71.94%	61.68%	60.93%	71.13%	62.78%	61.99%
Fit model criteria						
LL	− 4310.51	− 1623.10	− 1608.90	− 4366.33	− 1625.83	− 1610.60
AIC (−2LL)	8621.02	3246.20	3217.80	8732.66	3251.66	3221.20

*SE* Standard error, *ICC* Intraclass correlation coefficient, *LL* Log likelihood, *AIC* Akaike information criterion

## Discussion

ITNs are well-designed and stable malaria prevention and control strategy recommended in malaria-endemic regions globally [42]. They offer protection against malaria by deterring or killing mosquito vectors [42, 43]. ITNs distribution programs aim to provide and maintain ITN access and encourage consistent use, thereby ensuring anticipated impact on malaria control through prevention of transmission by mosquitoes [4, 5, 7–9, 13, 44, 45]. Despite increasing ITNs distribution efforts, the impact of socio-demographic disparities and factors influencing ITN ownership and use, particularly in rural communities remains unclear. This study evaluated socio-demographic determinants of ITNs ownership and use for malaria protection among individuals from rural communities in five districts of Mainland Tanzania.

The findings of these CSS which were conducted in 2023 showed that the overall ITNs ownership and use were 77.6 and 77.2%, respectively, but less than the national targets of reaching ownership of 80% by 2023 and 85% by 2025 [12]. However, these rates were relatively higher compared to previous findings reported in Tanzania and elsewhere [2, 46–48], with substantial variations across districts. The observed higher rates are probably the outcome of both routine and annual distribution campaigns that serve as keep-up strategy to sustain and increase ITNs access at both individual and household levels in Tanzania [9, 12, 45, 49]. Increased open ITNs retails has also contributed to the elevated access and use of bed nets among underserved groups in hard-to-reach areas [50, 51].

In the current study, females were 27% more likely to own and use ITNs compared to males. Similarly, under-fives showed higher ITNs access, with 86% and 2.26 times more likelihood of owning and using the nets, respectively, than adults. The higher likelihood of ITNs ownership and use in these groups is expected, as women and under-fives are given free nets through reproductive and child health (RCH) programs during their antenatal care and immunization visits in Tanzania, aiming at protecting these biologically vulnerable groups from malaria [45, 52, 53]. ITNs use among females and under-fives in the current study exceeded nationwide averages of 64 and 66% reported in the 2022 malaria indicator survey and other studies done in Tanzania [2, 48, 54]. This may also be attributed to the positive attitude fostered by ongoing BCC campaigns, which primarily target women and under-fives to encourage acquisition and consistent use of bed nets [15, 55].

Among all five districts covered by the CSS, three (Nyasa, Ludewa and Muheza) had notably higher odds (> 4.52) of both ITNs ownership and use, while Buhigwe showed 57 and 55% higher odds of ITNs ownership and use, compared to Kyerwa. Higher odds in Nyasa districts (Ruvuma) are linked to annual ITNs distribution rollout which has prioritized southern regions including Ruvuma as a perennial malaria endemic zone following the 2010/11 universal coverage campaign, plus subsequent SNPs which have been implemented in these regions to enhance ITNs access and sustain coverage [9, 11, 45]. The increased odds of ITNs ownership and use in other districts stem from targeted mass replacement campaigns of 2021 for Muheza (Tanga) and in both 2021 and 2023 for Ludewa district and other distribution channels, such as RCH and SNP designed to sustain ITNs access [45, 52]. Kyerwa demonstrated lowest ITNs ownership and use in this study, probably attributable to a lack of running distribution program in the surveyed villages. NMCP should prioritize targeted mass replacement to ensure ITNs access across all population groups in this district, alongside reviving SNPs for sustainable coverage amid recently reported high malaria-risk [29, 30, 56]

Education level was significantly associated with ITNs ownership and use, with notable gaps between participants who either completed primary education (were 60% and 55% more likely to own and use ITNs, respectively) or had secondary education and/or above (2.18 and 2.21 times more likely to own and use ITNs, respectively) compared to those with no formal education. These findings are congruent with studies done elsewhere [48, 57, 58]. However, a different pattern was observed in Ludewa, where ITNs ownership and use were higher among participants with no formal education compared to those with higher education. This finding appears counterintuitive given well-established association between education and uptake of preventive health behaviours [59–61] and is likely driven by small subgroup size, which may have resulted in unstable estimates. The higher odds among individuals with primary or higher education may be due to increased likelihood of having learned about malaria prevention in schools and their ability to read and understand malaria prevention messages disseminated through leaflets, radio or television [60, 62, 63]. Therefore, NMCP should strengthen ITNs distribution through strategic channels, such special groups ITNs distribution tailored and targeted health education for individuals with little or no formal education to boost their awareness on malaria prevention tools and reinforce the benefits of acquiring and using ITNs consistently.

As expected, the findings of the current study also showed that SES of the household was positively associated with ITNs possession and use (p < 0.001). Participants from households with high or moderate SES were more likely to own and use ITNs compared to those from households with low SES. This is similar to findings reported elsewhere in sub-Saharan Africa [64, 65] and is primarily influenced by financial constraints, which make individuals from low- and middle-income households to often have limited ability to afford ITNs and other malaria control interventions [13, 66, 67]. Therefore, government should consider increasing ITNs subsidies in both private and public ITNs distribution channels, including initiation and implementation of special voucher/coupon systems for financially disadvantaged groups to facilitate their access to ITNs [49, 68, 69].

Participants from non-farming sectors, including business, fishing, and formal employment exhibited higher ITNs ownership compared to farmers, which aligns with findings reported elsewhere [46, 70]. This can largely be attributed to relatively greater financial capacity that enables them to afford non-subsidized ITNs from commercial channels [46, 71]. Individuals from formal sectors, particularly those in education, health and community leadership, are well-informed about malaria and its prevention strategies through their education and qualified roles. They are also involved in malaria prevention initiatives, to raise community awareness and promote access to as well as use of ITNs [71, 72]. To address occupational disparities in ITNs ownership and use, tailored ITNs distribution strategies targeting specific occupational groups, particularly farmers, should be considered. Integrating ITNs distribution with community health workers outreach programs that engage farmers in their local settings may improve both ITNs access and consistent use [13, 63]. Additionally, provision of subsidized ITNs and embedding distribution programs into broader community health initiatives could enhance equitable coverage across all occupational sectors [65, 73].

Despite falling below under-fives in all districts except Muheza, school children consistently demonstrated high ITNs ownership and use, with overall ownership of 75.5% and use of 75.2%, which aligns with findings reported elsewhere [9, 29, 45]. This consistency is likely due to targeted ITNs distribution programs, such as SNPs that have utilized schools as channel for sustaining ITNs coverage in Tanzania since 2013 and scaled-up annually to ensure access and use of ITNs among school children [6, 8, 9, 11, 45, 74]. Nevertheless, school children in Tanzania continue to bear a significantly high malaria burden and this calls for further research to identify and address the underlying causes of their ongoing vulnerability [75–77]. The lower ITNs ownership and use in males is probably due to the lack of continuous ITNs distribution initiative targeting this group, particularly adult males [54, 78, 79]. Therefore, as NMCP strives for universal ITNs coverage, a thorough assessment of gender and age disparities is essential for informing better programs implementation in the future [54, 79, 80]. Males may also be less likely to sleep under ITNs due to misconceptions and false beliefs, including concerns that insecticide used in treated nets can affect fertility or impair male sexual performance [13, 81, 82]. Such perceptions may lead to some individuals to reject the freely distributed ITNs. Thus, strengthening social behaviours change communication strategies and implementing age-specific ITNs distribution approaches would address prevailing misconceptions and improve equitable ITNs access and consistent use among young adults.

This study had several limitations including the cross-sectional design that relied on participants’ willingness to enrol in the CSS and an exclusion of individuals who were out of their homes during the survey, which may have potentially introduced a selection bias through non-randomly sampling and underrepresentation, respectively. Reliance on self-reported data on ITNs use may also be influenced by recall bias or social appeal, leading to under or overestimation of actual practices [83]. However, ITNs use was assessed by whether the respondents slept under an ITN the previous night before the survey, a short recall period that minimizes recall bias and aligns with standard malaria indicator survey protocol [2, 84, 85]. Despite these limitations, the findings from this study provide valuable insights into ITNs ownership and use for malaria control in rural areas; and the consistency of these findings with prior studies conducted in Tanzania and elsewhere in sub-Saharan Africa suggests that selection bias was minimal, enhancing the generalizability of the results to similar endemic context.

## Conclusion

ITNs ownership and use were relatively high among rural residents in five districts. However, both fell short of the NMCP`s 80% target which was projected for 2023. Higher ownership and use were observed among females, under-five, participants with primary or higher education and those from high-SES households. These findings underscore persistent disparities in the current ITNs distribution programs and the need for targeted distribution strategies to achieve universal coverage across all population groups in Tanzania.

## Supplementary Information

Supplementary Material 1.

## Data Availability

Data supporting the findings of this study have been summarized and included in this paper, but more detailed data can be obtained from the corresponding author upon reasonable request, with institutional approval from NIMR and a signed data transfer agreement between NIMR and the recipient.

## Abbreviations

AIC: Akaike information criterion
BCC: Behaviour change and communication
CIs: Confidence intervals
CSS: Cross-sectional survey
ICC: Intracluster correlation coefficient
IDs: Identification numbers
ITNs: Insecticide-treated bed nets
IQR: Inter-quantile range
LL: Log likelihood
NIMR: National Institute for Medical Research
MSMT: Molecular Surveillance of Malaria in Mainland Tanzania
NMCP: National Malaria Control Programmme
ODK: Open Data Kit
PCA: Principal component analysis
PO-RALG: President’s Office, Regional Administration and Local Government
RCH: Reproductive and child health
SES: Socio-economic status
SNPs: School net programme
TNVS: Tanzania National Voucher Scheme
VIF: Variance inflation factor
WHO: World Health Organization
