# Supplementary material for: Socio-demographic predictors of insecticide-treated bed net ownership and utilization for protection against malaria by rural community members across five regions of Mainland Tanzania
Source: Malar J. 2026 May 7;25:252. doi: 10.1186/s12936-026-05926-9 (PMC13321573; doi:10.1186/s12936-026-05926-9)
Supplement: Supplementary file 1 — Supplementary Material 1. [file 12936_2026_5926_MOESM1_ESM.docx]

**Supplementary Table 1: Selected assets variables which were used to assess the socio-economic status, categories and their corresponding scores.**

| **Variable** | **Categories** | **SES score** |
| --- | --- | --- |
| Ownership of a radio | 0=No, 1=Yes | 0.3455 |
| Ownership of motorcycles | 0=No, 1=Yes | 0.3383 |
| Number of acres of land cultivated | 0=None, 1= 1-3=1 2=4 or more | 0.3379 |
| Possession of mobile phones | 0=No, 1=Yes | 0.3332 |
| Number of sleeping rooms/house size | Continuous (1-12) | 0.3198 |
| Source of light | 0 = local lamp [koroboi], 1 = kerosene lump/electricity) | 0.2928 |
| Occupation of head of HH- small business | 0=No, 1=Yes | 0.2224 |
| Toilet - wall type | 0 = Thatch/mud walls, 1 = Bricks) | 0.2155 |
| Source of cooking energy | 0 = Firewood, 1 = charcoal/kerosene/electricity) | 0.1904 |
| Number of sheep/goats | 0 = None, 1 = 1-10, 2 = More than 10) | 0.1902 |
| Possession of bicycles | 0=No, 1=Yes | 0.1422 |
| Number of chickens | 0=None, 1=1-20, 2 =21 or more | 0.1405 |
| Occupation of head of HH -formal employment | 0=No, 1=Yes | 0.1347 |
| Occupation of head of HH - livestock keeping | 0=No, 1=Yes | 0. 1231 |
| Number of cattle | 0=None, 1= 1-4, 2= 5 or more | 0.1126 |
| Ownership of a house | 0=No, 1=Yes | 0.0886 |
| Occupation of head of HH-fishery | 0=No, 1=Yes | 0.0804 |
| Source of drinking water | 1=River/Lake, 2=Tap water /closed well | 0.0028 |

*HH = household, SES = socio-economic status*

**Supplementary Table 2A: Bed net ownership among participants in the five surveyed districts**

| Variable | Ludewa | Buhigwe | Muheza | Kyerwa | Nyasa | Total |
| --- | --- | --- | --- | --- | --- | --- |
| Enrolled, N | 611 | 1453 | 1255 | 4454 | 2455 | 10228 |
| Bed net ownership, n (%) | 555 (90.8) | 1101 (75.8) | 1154 (92.0) | 2867 (64.4) | 2262 (92.1) | 7939 (77.6) |
| Sex, n (%) |  |  |  |  |  |  |
| Female | 330 (91.2) | 694 (75.9) | 683 (94.9) | 1743 (66.0) | 1414 (92.7) | 4864 (78.9) |
| Male | 225 (90.4) | 407 (75.5) | 471 (88.0) | 1124 (62.0) | 848 (91.3) | 3075 (75.7) |
| p-value | 0.737 | 0.857 | <0.001 | 0.006 | 0.218 | <0.001 |
| Age groups, n (%) |  |  |  |  |  |  |
| <5 years | 95 (99.0) | 215 (82.7) | 148 (92.5) | 618 (74.0) | 325 (96.2) | 1401 (83.0) |
| 5 - <15 years | 192 (87.7) | 522 (77.7) | 477 (96.8) | 819 (55.9) | 739 (94.1) | 2749 (75.5) |
| 15+ years | 268 (90.5) | 364 (69.9) | 529 (87.9) | 1430 (66.6) | 1198 (89.9) | 3789 (77.3) |
| p-value | 0.006 | <0.001 | <0.001 | <0.001 | <0.001 | <0.001 |
| History of fever - past 48 hours, n (%) |  |  |  |  |  |  |
| Yes | 64 (88.9) | 245 (72.5) | 216 (87.8) | 851 (63.4) | 95 (91.4) | 1471 (70.0) |
| No | 491 (91.1) | 856 (76.8) | 938 (93.0) | 2061 (64.8) | 2167 (92.2) | 6468 (79.6) |
| p-value | 0.542 | 0.107 | 0.008 | 0.382 | 0.759 | <0.001 |
| Fever at presentation (≥37.5°C), n (%) |  |  |  |  |  |  |
| Yes | 5 (100) | 20 (80) | 19 (90.5) | 85 (62.5) | 45 (88.2) | 174 (73.1) |
| No | 550 (90.8) | 1081 (75.7) | 1135 (92) | 2782 (64.4) | 2217 (92.2) | 7765 (77.7) |
| p-value | 0.476 | 0.619 | 0.802 | 0.644 | 0.295 | 0.091 |
| Education level, n (%) |  |  |  |  |  |  |
| None | 11 (91.7) | 53 (57.6) | 33 (82.5) | 337 (59.5) | 60 (80) | 494 (62.9) |
| Incomplete primary | 94 (87.8) | 277 (76.5) | 213 (94.7) | 498 (60.2) | 469 (92.5) | 1551 (76.5) |
| Completed primary | 184 (92.5) | 201 (75.6) | 233 (88.6) | 651 (71.9) | 706 (90.8) | 1975 (81.9) |
| Secondary or above | 18 (85.7) | 10 (100) | 27 (96.4) | 55 (73.3) | 84 (96.6) | 194 (87.8) |
| Studying | 62 (91.2) | 204 (77.0) | 123 (96.1) | 218 (56.5) | 215 (92.7) | 822 (76.2) |
| p-value | 0.662 | 0.001 | 0.005 | <0.001 | 0.002 | <0.001 |
| Occupation, n (%) |  |  |  |  |  |  |
| Farmer | 213 (91.8) | 45 (75.0) | 5 (83.3) | 1108 (67.8) | 575 (90.8) | 1946 (75.9) |
| Students | 137 (88.4) | 108 (78.8) | 29 (90.6) | 604 (56.9) | 446 (95.1) | 1324 (71.4) |
| Children | 137 (93.2) | 125 (78.1) | 47 (90.4) | 780 (66.1) | 294 (96.4) | 1383 (75.0) |
| Others | 19 (95.0) | 14 (82.4) | 8 (100) | 47 (73.4) | 71 (95.6) | 159 (86.9) |
| p-value | 0.431 | 0.906 | 0.747 | <0.001 | 0.002 | <0.001 |
| Family size |  |  |  |  |  |  |
| <4 people | 138 (93.88) | 57 (61,96) | 120 (92.31) | 537 (64.31) | 531 (91.44) | 1365 (77.34) |
| ≥4 people | 401 (89.71) | 774 (76.56) | 1002 (92.01) | 2117 (64.33) | 1515 (92.55) | 5809 (77.71) |
| p-value | 0.103 | 0.002 | 0.906 | 0.993 | 0.398 | 0.734 |
| Socio-economic status, n (%) |  |  |  |  |  |  |
| High | 237 (93.7) | 464 (77.6) | 768 (92.2) | 768 (69.7) | 1090 (93.4) | 3327 (77.9) |
| Moderate | 252 (87.8) | 337 (76.8) | 255 (93.1) | 993 (65.5) | 732 (93.1) | 2569 (77.8) |
| Low | 50 (92.6) | 197 (68.9) | 109 (87.9) | 894 (59.2) | 378 (86.7) | 1628 (67.6) |
| p-value | 0.056 | 0.014 | 0.194 | <0.001 | <0.001 | <0.001 |

*N=Total participants, n=Number of observed participants, °C=degree Celsius*

**Supplementary Table 2B: Bed net use among participants across the five selected districts**

| Variable | Ludewa | Buhigwe | Muheza | Kyerwa | Nyasa | Total |
| --- | --- | --- | --- | --- | --- | --- |
| Enrolled, N | 611 | 1453 | 1255 | 4454 | 2455 | 10228 |
| Bed net use, n (%) | 555 (90.8) | 1094 (75.3) | 1145 (91.2) | 2849 (64.0) | 2256 (91.9) | 7899 (77.2) |
| Sex, n (%) |  |  |  |  |  |  |
| Female | 330 (91.2) | 689 (75.4) | 680 (94.4) | 1734 (65.7) | 1409 (92.3) | 4842 (78.6) |
| Male | 225 (90.4) | 405 (75.5) | 465 (86.9) | 1115 (61.5) | 847 (91.2) | 3057 (75.2) |
| p-value | 0.737 | 0.917 | <0.001 | 0.005 | 0.307 | <0.001 |
| Age groups, n (%) |  |  |  |  |  |  |
| <5 years | 95 (99.0) | 214 (82.3) | 148 (92.5) | 616 (73.7) | 325 (96.2) | 1398 (82.7) |
| 5 - <15 years | 192 (87.7) | 518 (77.1) | 473 (95.9) | 815 (55.4) | 738 (94.0) | 2736 (75.2) |
| 15+ years | 268 (90.5) | 362 (69.5) | 524 (87.0) | 1418 (66.0) | 1193 (89.6) | 3765 (76.9) |
| p-value | 0.006 | <0.001 | <0.001 | <0.001 | <0.001 | <0.001 |
| History of fever past 48 hours, n (%) |  |  |  |  |  |  |
| Yes | 64 (88.9) | 243 (71.9) | 213 (86.6) | 850 (63.4) | 94 (90.4) | 1464 (69.7) |
| No | 491 (91.1) | 851 (76.3) | 932 (92.4) | 1999 (64.2) | 2162 (92.0) | 6435 (79.2) |
| p-value | 0.542 | 0.098 | 0.004 | 0.567 | 0.564 | <0.001 |
| Fever at presentation (≥37.5°C), n (%) |  |  |  |  |  |  |
| Yes | 5 (100) | 20 (80) | 19 (90.5) | 84 (61.8) | 44 (86.3) | 172 (72.3) |
| No | 550 (90.8) | 1074 (75.2) | 1126 (91.3) | 2765 (64.0) | 2212 (92.0) | 7727 (77.4) |
| p-value | 0.476 | 0.582 | 0.901 | 0.587 | 0.137 | 0.065 |
| Education level, n (%) |  |  |  |  |  |  |
| None | 11 (91.7) | 53 (57.6) | 33 (82.5) | 336 (59.4) | 59 (78.7) | 492 (62.7) |
| Incomplete primary | 94 (87.9) | 274 (75.7) | 209 (92.9) | 491 (59.4) | 468 (92.3) | 1536 (75.7) |
| Completed primary | 184 (92.5) | 200 (75.2) | 231 (87.8) | 645 (71.1) | 702 (90.2) | 1960 (81.3) |
| Secondary or above | 18 (85.7) | 10 (100) | 27 (96.4) | 55 (73.3) | 84 (96.6) | 194 (87.8) |
| Studying | 62 (91.2) | 204 (77.0) | 122 (95.3) | 218 (56.5) | 215 (92.7) | 821 (76.1) |
| p-value | 0.662 | 0.001 | 0.021 | <0.001 | 0.001 | <0.001 |
| Occupation, n (%) |  |  |  |  |  |  |
| Farmer | 213 (91.8) | 45 (75.0) | 5 (83.3) | 1099 (67.3) | 574 (90.7) | 1936 (75.5) |
| Students | 137 (88.4) | 106 (77.4) | 29 (90.6) | 589 (56.4) | 446 (95.1) | 1316 (71.0) |
| Children | 137 (93.2) | 125 (78.1) | 47 (90.4) | 778 (65.9) | 294 (96.4) | 1381 (74.9) |
| Others | 19 (95.0) | 14 (82.4) | 8 (100) | 46 (71.9) | 70 (94.6) | 157 (85.8) |
| p-value | 0.431 | 0.923 | 0.747 | <0.001 | 0.002 | <0.001 |
| Family size |  |  |  |  |  |  |
| <4 people | 138 (93.88) | 57 (61.96) | 119 (91.54) | 529 (63.35) | 513 (91.44) | 1356 (76.83) |
| ≥4 people | 401 (89.71) | 769 (76.06) | 994 (91.28) | 2107 (64.02) | 1509 (92.18) | 5780 (77.32) |
| p-value | 0.130 | 0.003 | 0.902 | 0.719 | 0.579 | 0.654 |
| Socio-economic status, n (%) |  |  |  |  |  |  |
| High | 237 (93.7) | 461 (77.1) | 763 (91.6) | 765 (69.4) | 1086 (93.1) | 3312 (83.8) |
| Moderate | 252 (87.8) | 334 (76.1) | 252 (92.0) | 987 (65.1) | 730 (92.9) | 2555 (77.4) |
| Low | 50 (92.6) | 197 (68.9) | 108 (87.1) | 885 (58.7) | 378 (86.7) | 1618 (67.2) |
| p-value | 0.056 | 0.025 | 0.226 | <0.001 | <0.001 | <0.001 |

*N=Total participants, n=Number of observed participants, °C=degree Celsius*
